# Immune Response to Lipoproteins in Atherosclerosis

**DOI:** 10.1155/2012/571846

**Published:** 2012-08-23

**Authors:** Sonia Samson, Lakshmi Mundkur, Vijay V. Kakkar

**Affiliations:** ^1^Department of Molecular Immunology, Thrombosis Research Institute, Narayana Hrudayalaya Hospital, 258/A, Bommasandra Industrial Area, Bangalore 560099, India; ^2^Scientific Chairman, Thrombosis Research Institute, London, UK

## Abstract

Atherosclerosis, the underlying cause of cardiovascular disease, is characterized by chronic inflammation and altered immune response. Cholesterol is a well-known risk factor associated with the development of cardiovascular diseases. Elevated serum cholesterol is unique because it can lead to development of atherosclerosis in animals and humans even in the absence of other risk factors. Modifications of low-density lipoproteins mediated by oxidation, enzymatic degradation, and aggregation result in changes in their function and activate both innate and adaptive immune system. Oxidized low-density lipoprotein (LDL) has been identified as one of the most important autoantigens in atherosclerosis. This escape from self-tolerance is dependent on the formation of oxidized phospholipids. The emerging understanding of the importance of immune responses against oxidized LDL in atherosclerosis has focused attention on the possibility of development of novel therapy for atherosclerosis. This review provides an overview of immune response to lipoproteins and the fascinating possibility of developing an immunomodulatory therapy for atherosclerosis.

## 1. Introduction

Cardiovascular diseases remain the leading cause of global morbidity and mortality. As per the WHO estimates 17.3 million people died of CVD in 2008 representing almost 30% of global mortality. It is estimated that this number will rise to 23.6 million by 2030 with almost 80% of the death occurring in low and middle income countries. The most important risk factors of heart disease and stroke are unhealthy diet, physical inactivity, tobacco use, and harmful use of alcohol. These result in raised blood pressure, raised levels of glucose and lipids in blood, overweight, and obesity which constitute the metabolic syndrome [1].

Higher level of cholesterol in blood has traditionally been considered as established risk factors for CVD. However, increased total cholesterol concentrations in plasma do not accurately predict the risk of coronary heart disease as it includes the sum of all cholesterol carried not only by atherogenic lipoproteins, that is, very low-density lipoprotein [VLDL], low-density lipoprotein [LDL], and intermediate-density lipoprotein [IDL], but also by antiatherogenic lipoproteins, that is, high-density lipoprotein, [HDL]. It is also known that the small, dense LDL cholesterol is more atherogenic than large, buoyant particles, and oxidation of LDL increases its atherogenicity. The relationship between LDL cholesterol and risk for CVD is well established, and measurement of LDL is routinely used for risk assessment, as well as risk management [2]. Over the last four decades, significant progress has been made towards the prevention of CVD, primarily by the use of statins which result in lowering the cholesterol levels. However, the increasing epidemic of metabolic syndrome and Type 2 diabetes mellitus (T2DM) has slown down this progress. Although the use of statins has accounted for the significant reduction in the morbidity and mortality associated with CVD, the risk is not completely eliminated despite effective lipid-lowering treatment [3]. It is estimated that the current therapies prevent only 30% of clinical events, suggesting an urgent need for newer therapeutic strategies [3].

For many years atherosclerosis was believed to be a disease of lipid accumulation in the vessel wall. Extensive research on the pathophysiology of the disease has brought about a paradigm shift in our understanding of CVD, and atherosclerosis is now accepted as a multifactorial, multiphase chronic inflammatory disease with immunological activity at every stage, from initiation to progression and plaque rupture [4–6]. This review will concentrate on immune response to lipoproteins, its role in the development of atherosclerosis, and modulation of immune response to lipoprotein as therapeutic strategy.

## 2. Immune Response and Atherosclerosis

**Figure 1:**
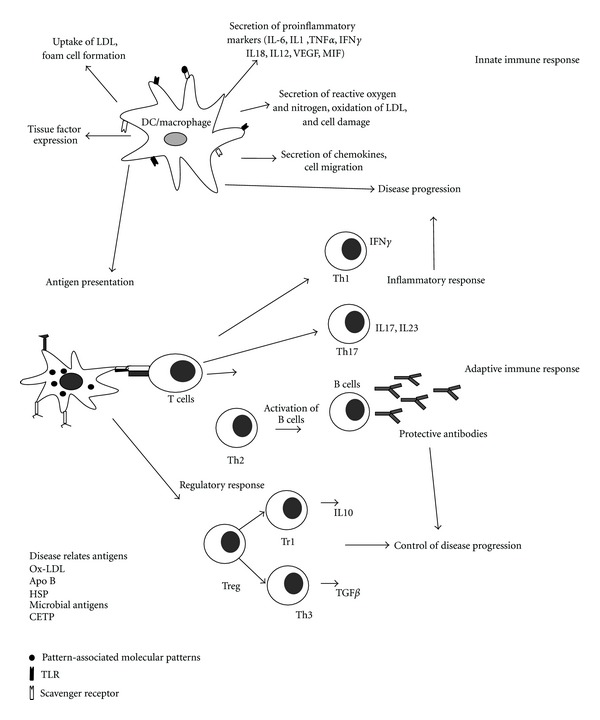
Immune response in atherosclerosis. Macrophages and dendritic cells are the important components of innate immune response in atherosclerosis. Uptake of modified LDL particles such as oxLDL through scavenger receptors leads to the intracellular accumulation of cholesterol leading to activation and expression of a series of genes encoding proinflammatory molecules, including cytokines, chemokines, eicosanoids, proteases, oxidases, and costimulatory molecules. Adaptive immune response is activated when self-antigens, oxidized LDL, and other disease-related antigens are presented to T cells by the macrophages/dendritic cells. The concomitant release of cytokines determines the maturation of the T cell recognizing the antigen. Differentiation into Th1 cells results in inflammatory response, while Th2 leads to activation of antigen-specific B cells. These B cells produce antibodies to the disease-specific antigens. Regulatory T cells induce a tolerogenic response mediated by Tr1 cells and Th3 cells which secrete IL10 and TGF-*β* respectively, which inhibit the progression of the disease. Th, T helper cells; Treg, regulatory T cell; IL, interleukin; VEGF, vascular endothelial growth factor; TNF, tumor necrosis factor; MIF, migration inhibition factor; Ox-LDL, oxidized low-density lipoprotein; HSP, heat shock protein, CETP, cholesteryl ester transfer protein.

Atherosclerosis, which manifests itself as acute coronary syndrome, stroke, and peripheral arterial diseases, is a chronic inflammatory disease of the arterial wall [7]. Immune system plays an important role in the development, progression, and the complications associated with atherosclerosis [5]. Both innate and adaptive immune responses are associated with the progression of the disease (Figure 1). The retention of cholesterol in the subendothelial region of the vessel is the central pathogenic event that starts the atherosclerotic lesion formation [8]. Lipids, such as cholesterol and triglycerides, are insoluble in plasma and are carried by lipoproteins that transport them to various tissues, and LDL is normally associated with the apolipoprotein (Apo) B-100. An increase in plasma LDL levels leads to an increased rate of its entry into the intima, and consequently a higher level of LDL is observed in the intimal region [9]. The interaction of positively charged ApoB to negatively charged proteoglycans leads to the retention of ApoB-linked lipoproteins in the vessel wall [10]. These sequestered lipoproteins are susceptible to modification by oxidation, enzymatic cleavage, and aggregation [11]. Immune response to these modified lipoproteins drives the pathogenic evolution of the plaque by releasing proinflammatory mediators leading to a chronic inflammatory reaction. Oxidized LDL induces the formation of foam cells and fatty streaks in the vessel wall which is the hallmark of initiation of atherosclerosis [12]. Macrophages from the host immune system try to clean up cholesterol deposits in arteries, but once they are loaded with the unhealthy form of cholesterol, they get stuck in the arteries, triggering the body's inflammatory response. These cholesterol-loaded macrophages line the artery wall and become major components of the growing plaque. As the atherosclerotic lesion evolves, other immune inflammatory cells such as T cells, dendritic cells, and mast cells accumulate in the region. Macrophages and dendritic cells are known to contribute to the innate immune response by generating free oxygen radicals, proteases, complement factors and cytokines. Macrophages also produce chemokines including the T-cell attractant CCL5 (RANTES) to attract other immune cells into the growing plaque [13, 14]. A fibrous cap of variable thickness, composed mainly of collagen, covers the lesion, while the shoulder region consists of activated T cells, macrophages, and mast cells [7]. The early fatty streaks develop into a complex lesion consisting of apoptotic and necrotic cells, cell debris, and cholesterol crystals which form the necrotic core over a long period of time [15]. Adaptive immunity recognizes specific epitopes on antigens that are processed and presented by the antigen-presenting cells to the T cells, leading to lymphocyte activation and secretion of cytokines and antigen-specific antibodies. T cells reacting to disease-related antigens such as Ox-LDL, HSP60, bacterial, and viral antigens have been found in the human lesions [16–18] (Figure 1).

## 3. ProAtherogenic and Atheroprotective Immune Response

Four major subsets of T helper cells are involved in the adaptive immune response: helper T-cell subsets Th1 and Th2, regulatory T cells, and the Th17 cells. The CD8^+^ T cells promote atherogenesis when activated by foreign antigens, but their precise role in atherogenesis remains unclear as their depletion is not associated with any change in the lesion formation [19]. Th1 cells the induce activation of macrophages, neutrophils, and cytotoxic T lymphocytes and secrete proinflammatory cytokines such as interferon *γ* and IL12. They are more prevalent in the lesions of both human and ApoE^−/−^ mice suggesting that atherosclerosis is a Th1-dominant disease [20, 21]. Th2 cells are involved in allergic diseases such as atopic allergy and asthma [22]. Their role in atherosclerosis seems to depend on the stage and site of the disease. Increased concentrations of Th2 cytokines in lymphoid organs and atheroprotective anti-oxLDL IgM antibodies in the serum resulted in a significant reduction in atherosclerotic plaque development [23]. However in mice deficient in IL4, the archetypical Th2 cytokine, atherosclerosis was less severe than in IL4-sufficient mice [24]. Th17 cells produce the proinflammatory cytokine IL17 and promote autoimmune diseases [25, 26]. Significant increase in peripheral Th17 cells, cytokines IL17, IL6 and IL23, and transcription factor ROR*γ* levels was reported in patients with acute coronary syndrome (ACS) when compared to control [27]. A functional imbalance between the Th17/Treg was also reported in patients with acute coronary syndrome (ACS), suggesting a potential role for these cells in plaque destabilization and the onset of ACS.

Treg cells are a subpopulation of T cells specialized in maintaining immune homeostasis and self-tolerance by suppressing pathogenic immune responses. Treg cells are heterogeneous and can be subdivided schematically into two major subsets: natural (n Treg) and induced (i Treg). These cells are characterized by expression of CD25 (a subunit of the IL2 receptor) and CD4, on the surface and intracellular expression of the transcription factor fork head box protein P3 (FoxP3) [28]. Treg cells can inhibit effector T cells by contact-dependent suppression of cell proliferation and downregulate the availability of growth factors to effector T cells by enhanced consumption of IL2, and by inhibiting the effector cell functions through secretion of the anti-inflammatory cytokines TGF-*β*, IL10, and IL35 [29]. The clinical manifestation of atherosclerosis can be linked to inflammation mediated by the Th1 cells, while Treg cells may be involved in the stabilization of disease. Several review articles have discussed the role of immune response in atherosclerosis in detail [5, 7, 27, 30–41].

## 4. Immune Response to Lipoproteins in Atherosclerosis

The positive role of lipoproteins in the development of cardiovascular diseases has been reported by several epidemiological studies so much so that atherosclerosis was considered a lipid-mediated disease. A key early step in atherogenesis is the formation of the fatty streak, consisting of a subendothelial collection of foam cells, which are cholesterol-laden macrophages or smooth muscle cells [42]. LDL does not trigger an immune response in its native state. Formation of oxidized phospholipids and aldehyde-modified breakdown fragments of apolipoprotein B-100 (apoB-100) exposes neoantigens which cause a breakdown in self-tolerance and induces inflammatory reactions [43, 44]. Oxidation of LDL generates reactive aldehydes and truncated lipids by cleaving the fatty acid double bonds in phospholipids, triglycerides, and cholesteryl esters [45]. Modified phospholipids such as lysophosphatidyl choline and trimethylamine N-oxide can trigger potent immune response by activating NKT cells, macrophages, and endothelial cells [15, 46, 47]. Oxidative modification of ApoB 100 also causes degradation of ApoB and release of small peptides which increase vascular permeability [48, 49]. Accumulation of monocytes/macrophages, smooth muscle cells, and T cells within the arterial wall in response to proinflammatory molecules constitutes a hallmark of developing plaque [5]. Oxidized LDL (OxLDL) has been identified as one of the most important autoantigens in atherosclerosis. The Activation of both innate and adaptive immune responses against OxLDL is the major cause of inflammation and its pathological consequences [31, 36, 50, 51]. Modified LDL interact with scavenger receptors while OX LDL binds to CD36 receptor on monocytes and macrophages and form foam cells [44]. Foam cells are highly immunogenic and attract the adhesion, migration, and activation of the cells of the immune system thus contributing to the development of the disease.

Antigen-presenting cells take up modified LDL and initiate an adaptive immune response by presenting these antigens to T cells, which proliferate to amplify the immune response [52]. Upon renewed exposure to the specific antigen, these T cells produce cytokines and trigger inflammation.

**Figure 2:**
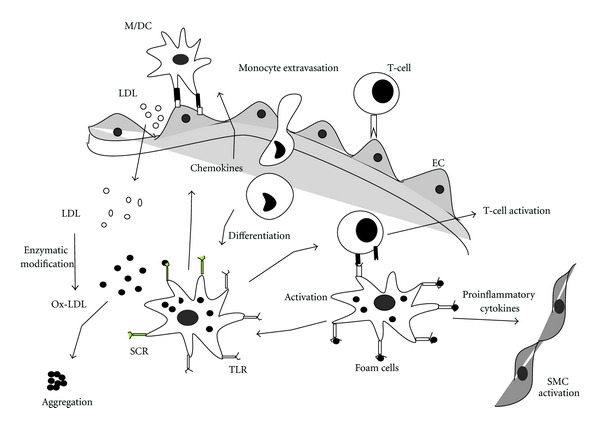
Immune response to LDL in atherosclerosis. The low-density lipoprotein (LDL) in the blood diffuses into the intima of the-vessel wall, where it gets oxidized by enzymes or reactive oxygen to form OxLDL. Modified LDL particles are taken up by macrophages that accumulate cholesterol and become foam cells. Ox-LDL causes overexpression of VCAM-1 and ICAM-1 by the endothelial cells, which attracts the monocytes, and T cells move into the vessel wall. Activated macrophages secrete proinflammatory mediators, such as TNF*α*, IL-1, MCP-1, and proteolytic enzymes (MMPs). Foam cells can process and present ApoB100 peptides to CD4+ T helper cells via MHC class II molecules. Antigen presentation to CD4+ TH1 cells triggers their activation, with ensuing release of IFN- and TNF known to have proatherogenic properties. VCAM, vascular cell adhesion molecule; ICAM, intercellular cell adhesion molecule; TNF, tumour necrosis factor; IL, interleukin; MCP, macrophage chemoattractant protein; MMP, matrix metalloproteinase, Th, T helper cells; ApoB, apolipoprotein B.

OxLDL is frequently present in sera of patients with coronary syndrome [53, 54] and also accumulates in atherosclerotic plaques [55]. Apart from the formation of foam cells, OxLDL exhibits a range of proatherogenic properties. It acts as a chemoattractant for circulating monocytes and can stimulate secretion of monocyte chemoattractant protein-1 by endothelial cells [56]. It is cytotoxic for endothelial cells cultured in serum-free medium, induces expression of macrophage colony stimulating factor, promotes the differentiation of monocytes to macrophages, attracts T cells into the growing atherosclerotic plaque, induces a wide variety of proinflammatory cytokines in macrophages, increases expression of vascular cell adhesion molecule-1 and is also immunogenic [57, 58]. The existence of a preexisting, natural immune response against oxidized LDL phospholipids mediated by IgM produced by B-1-cells has also been identified [43]. Immune complexes formed by modified LDL and corresponding antibodies are potent macrophage activators and direct overexpression of MHC-II, costimulatory molecules, and proinflammatory markers, thus creating an ideal conditions for Th1 activation [56–58]. Activated macrophages also release reactive oxygen radicals, enhancing the opportunity for LDL modification [5] which increases the immunogenic load, induce a more vigorous antibody response, and increase the formation of LDL immune complexes (Figure 2).

## 5. Natural Antibodies to Lipoproteins

Natural antibodies are defined as antibodies that are found in normal individuals in the complete absence of any exogenous antigenic stimulation and provide first line of defense against invading pathogens [59]. These antibodies bind to a number of self-antigens such as cell membrane components (phosphtidyl choline, glycolipids, etc.) [59, 60] single-stranded DNA [61] and cell surface molecules on T-cells such as Thy1 [62]. Many of these self-epitopes are also present on pathogens [63–65]. These natural antibodies are also termed as poly reactive to explain their cross reactivity with multiple self-and- non-self-antigens and are required for the immediate recognition and protection against invading pathogens [59, 66]. Natural antibodies are also involved in the removal of aging and dead cells, their debris, and self-antigens and thus protect from autoimmunity [32]. This role of protection from autoimmunity is very relevant especially under certain pathological conditions that involve increased accumulation of self-antigens such as oxidation specific epitopes during atherosclerosis [31].

Natural IgM antibodies are predominantly produced by a small subset of long-lived, self-replenishing B cells, termed B-1 cells which exhibit a conserved repertoire [65]. These antibodies are encoded in the germline genome and are not dependent on immunoglobulin gene rearrangement. They have broad specificities, but display low affinities and do not require T-cell stimulation of the B lymphocytes to produce antibodies. Presence of autoantibodies to epitopes of copper-oxidized LDL (Cu-OxLDL) and malondialdehyde-modified LDL (MDA-LDL) has been reported in human and animal models of atherosclerosis [67–69]. Cholesterol-fed ApoE^−/−^ mice were found to have very high autoantibody titers, particularly IgM, to a wide variety of Ox LDL epitopes [70]. B-cell hybridomas generated from these mice revealed that most of these autoantibodies were of IgM isotype, recognized the lipid and ApoB moieties of OxLDL, but not of native LDL, and had a specific recognition for phosphorylcholine group [71, 72]. The prototypic and best-characterized antibody against OxLDL, EO6, is identical to T15, a natural antibody known to recognize phosphorylcholine (PC) expressed as a capsular epitopes on *Streptococcus pneumonia *[43] and could also block OxLDL uptake by macrophages. Binder et al. identified the functional role of antiphospholipid antibodies in atherosclerosis by immunizing LDLr^−/−^ mice with heat-inactivated PC containing pneumococci [73]. The pneumococcal immunization was found to induce high titers of anti-OxLDL IgM (predominantly of the T15 clonotype) and significantly reduce atherosclerotic lesion in the aortic sinus. The uptake of OxLDL by macrophages was found to be inhibited by the plasma of immunized mice. In a similar attempt to use PC as a vaccine for atherosclerosis, Caligiuri et al. immunized mice with PC covalently linked to a carrier protein, keyhole limpet hemocyanin (KLH). Immunized mice showed a 40% reduction in lesions compared to control and sera from PC-KLH-immunized mice decreased, the uptake of OxLDL compared to sera of PBS-immunized mice [74]. Further studies in human revealed that patients recovering from pneumococcal infections contain IgM antibodies to the bacterial polysaccharide that significantly correlated with levels of anti-OxLDL IgM antibodies in the same serum sample. These findings suggest that PC-specific cross-reacting IgMs are also present in humans. Human IgG1 against a specific OxLDL epitope was reported to induce rapid and substantial regression of atherosclerotic lesions, possibly by stimulating lipid efflux and inhibiting macrophage recruitment [75]. These recombinant human atheroprotective antibodies could, thus, represent a novel strategy for rapid regression/stabilization of atherosclerotic lesions.

The mechanism of protection afforded by these antibodies has not been studied in detail so far [76, 77]. Binding of OxLDL by IgM antibodies could potentially neutralize most of their proinflammatory properties, which promote atherogenesis. The formation of circulating immune complexes of these IgMs with OxLDL may have protective properties by preventing LDL from entering vulnerable sites of the artery wall. A number of in vitro studies have suggested that these IgM antibodies block the uptake of OxLDL by macrophages and thus could prevent foam cell formation in vivo [73]. They could prevent the activation of endothelial cells and monocyte binding by apoptotic cells containing oxidized lipids [67]. It is still not clear whether passive therapy with these antibodies alone would be protective. Passive transfer of antiphosphorylcholine monoclonal antibodies reduced atherosclerosis supporting the protective role of natural antibodies [78]. Thus, a number of protective mechanisms have been suggested for natural antibodies; however, the relevance of these mechanisms in vivo is still not very clear.

 On the other hand existence of proatherogenic natural antibodies is also a possibility which has not been studied in detail so far, as some B-1 cell-derived IgMs have been shown to play a pathogenic role in intestinal ischemia/reperfusion injury [79]. Understanding the role of natural antibodies in health disease and autoimmunity is likely to open up novel therapeutic approaches for the control of atherosclerosis.

## 6. Antilipoprotein Antibodies: Friend or Foe?

**Figure 3:**
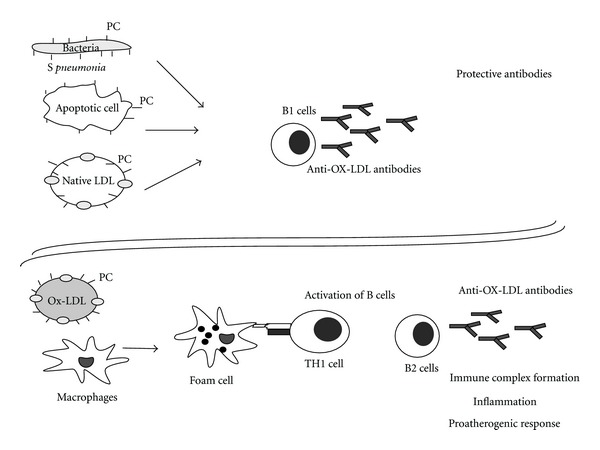
Protective and pathogenic antilipoprotein antibodies. Molecular mimicry occurs when LDL or a viable cell undergoes limited oxidative modification to present phosphocholine (PC) antigens like those expressed on bacteria (*Streptococcus pneumonia)*.These antigens stimulate B1 lymphocytes to produce the protective EO6/T15 autoantibodies. These antibodies are likely to block the uptake of Ox-LDL through scavenger receptors, promote the clearance of apoptotic cells, or neutralize the infecting bacteria resulting in a protective response. Extensive oxidation of LDL results in its conversion to Ox-LDL is taken up by macrophages and stimulate the T-cell-dependent immune response to produce IgG antibodies. These antibodies, along with other proinflammatory cytokines produced by foam cells, in turn accelerate lesion progression.

Antibodies to lipoproteins are exemplary in having both proatherogenic as well as protective function against atherosclerosis (Figure 3).

### 6.1. Pathogenic Effects of Anti-OxLDL Antibodies

OxLDL frequently presents in the sera of patients with coronary artery disease, and the serum concentrations of circulating oxLDL may correlate with the severity of CAD and acute coronary syndrome [53, 54, 80, 81]. Analysis of several studies suggests that, in humans, the humoral immune response to modified LDL is pathogenic. Adaptive response is known to generate IgG antibodies, and the predominance of IgG over IgM antibodies favors the formation of IgG-containing immune complexes with proinflammatory properties. Immune complexes formed with modified LDL and IgG antibodies have been shown to have significantly stronger proatherogenic and proinflammatory properties than modified LDL itself [82–85]. Atherosclerotic lesions also contain immunoglobulins that specifically recognize OxLDL [86], and these antibodies are believed to be the most effective parameters for predicting the extent of coronary atherosclerosis [82]. Their presence is also associated with a higher risk for coronary restenosis after coronary angioplasty [83].

Elevated levels of anti-Ox-LDL antibody are related to hypertension, systemic vacuities, peripheral arterial disease, endothelial dysfunction, atherosclerosis, and cardiovascular disease [69, 84, 85, 87–90]. Anti-OxLDL antibodies can also induce other effects, such as complement activation, and induction of adaptive immune response leading to inflammation. Different subclasses of anti-Ox LDL antibodies with a range of pathogenic effects are reported in humans [91]. IgG1 and IgG3 antibodies have been defined as proinflammatory, based on their ability to activate the complement system by the classical pathway and to interact with Fc*γ* receptors in phagocytic cells [68]. The involvement of IgG1 and IgG3 antibodies in immune complex disease is also well recognized [92]. However, there are reports showing negative or no correlation between anti-LDL antibodies and atherosclerosis [93, 94]. The measurement of free circulating autoantibodies depends on the magnitude of the antibody response, antibody avidity, and on the amount of antigen present in circulation. Soluble immune complexes are formed by the high-avidity antibodies and circulating Ox-LDL leading to inaccurate estimation of anti-Ox-LDL antibodies in the serum [52, 95].

### 6.2. Protective Effects of Ox-LDL Antibodies

Anti-Ox-LDL antibodies are present in healthy individuals as well as in patients with atherosclerosis [69, 96]. Several experimental studies in animals using Ox-LDL immunization have shown a positive correlation between high titers of anti-Ox-LDL antibodies and the degree of protection against atherosclerosis [97–100]. Transfer of B cells from atherosclerotic apolipoprotein (Apo) E- knockout mice (ApoE−/−) to young, ApoE−/− mice protected the latter from developing advanced disease [96]. Passive administration of recombinant human antibodies against aldehyde-modified apolipoprotein B-100 peptide sequences was observed to inhibit atherosclerosis in ApoE−/− mice [101]. These antibodies were found to modulate the development of fatty streaks as well as their progression to atherosclerotic plaque [102]. Anti-Ox-LDL antibodies are present in healthy individuals as well as in patients with atherosclerosis [69, 96]. Antibodies to Ox LDL seem to play an important role in regulating the level of OX LDL in human. Circulating antibodies recognizing ox-LDL have been found in children with no risk of CVD, and an inverse correlation was observed between plasma Ox-LDL concentrations with the levels of anti-Ox-LDL antibodies in healthy subjects [103, 104]. In another study anti-Ox-LDL antibody levels were inversely related to the intima-media thickness of the carotid arteries in a healthy population with no clinical signs of atherosclerosis [105]. These studies support the protective function of anti LDL antibodies in atherosclerosis which seem to be native antibodies that neutralize Ox-LDL [104, 105].

These observations raise several pertinent questions; is it possible that different epitopes on Ox-LDL determine its protection or pathogenicity? “Can we use anti-Ox-LDL antibodies for atheroprotection? Can OX-LDL be used as an immunogen for modulation of immune response without having any serious adverse effects? Careful consideration of these aspects will be most essential before they can be taken as candidates for therapeutic intervention.

## 7. Vaccine against Atherosclerosis

Over the last few years, considerable efforts have been made to develop a vaccine using epitopes from lipoproteins and heat shock proteins [106–110]. Considering atherosclerosis as an autoimmune disease wherein an immune response is triggered against the autoantigens, a vaccine which can restore the tolerance to these antigens would be effective in reducing the inflammatory response. Antigen-specific immune modulation is an attractive approach to prevent chronic inflammatory diseases without affecting the normal immune function of the host. Two self-antigens which have emerged as most important ones are related to LDL and heat shock protein (HSP) [60]. Normally T cells reacting to these antigens should be eliminated by negative selection, in the thymus leading to central tolerance. If oxidation of LDL generates neoantigens, all the T cell clones reactive to these would not be removed during thymic education [15]. Similarly in the case of HSP60, molecular mimicry between HSPs of pathogens and human could trigger autoimmune response leading to chronic inflammation. Peripheral tolerance plays a role in maintaining an immune homeostasis to these self-antigens under normal circumstances. Vaccines against atherosclerosis that are being currently developed are different from traditional vaccines for infectious diseases. An ideal vaccine is aimed at restoring the self-tolerance to autoantigens like LDL and heat shock proteins, reducing the inflammation, and balancing the pro- and anti-atherogenic immune response [111].

## 8. Modulation of Immune Response to Lipoproteins

Lipoprotein oxidation and subsequent formation of several new antigenic epitopes renders this molecule highly immunogenic, leading to both humoral and cellular immune response. Moreover LDL is a complex particle composed of a high-molecular-weight protein, apolipoprotein B-100 (ApoB100), neutral and polar lipids, and lipophilic antioxidants. Since the different epitopes of ox-LDL induce atherogenic immune responses, it is an attractive candidate to explore immune modulation. Immunization against Ox-LDL has been shown to reduce atherosclerosis by a number of studies [73, 75, 97, 101, 109, 112–115].

Immunization of hypercholesterolemic rabbits and LDLr^−/−^ mice with both MDA-LDL and Cu-Ox-LDL were found to generate high titers of antibodies and inhibit atherosclerosis development by 40–70%, suggesting an induction of atheroprotective immune response [68, 97, 100, 108]. The mechanism of protection afforded by this immunization is thought to be through IgM antibodies generated against Ox-LDL that are shown to block the uptake of Ox-LDL by CD36 receptors on macrophages, thus reducing foam cell formation [116]. Induction of oral tolerance to Cu-Ox-LDL and MDA-LDL mediated by Treg cells and TGF-*β* was also reported to attenuate the initiation and progression of atherosclerosis in LDLr^−/−^ mice [109]. Similar effects were also observed on tolerance induction to HSP60 [110].

The protein sequence of ApoB-100 was studied by Fredrikson et al. to identify potential antigenic epitopes that could have atheroprotective effects [112]. Out of the 302 overlapping 20 amino acid peptides synthesized, few were found to provoke an atheroprotective immune response in hypercholesterolemic mice [117]. Peptide 45, corresponding to the amino acid sequence 661–680 of ApoB100 was found to be one of the most effective atheroprotective peptides [113]. The presence of autoantibodies against this peptide sequence was also shown to be associated with reduced risk for development of myocardial infarction (MI) in humans [118]. Adoptive transfer of splenocytes from immunized mice as well as monoclonal antibody against this peptide was found to passively transfer protection [101]. Treatment with human recombinant IgG1 antibodies against the same epitope ameliorated the existing atherosclerotic lesions in ApoB^−/−^ and LDLr^−/−^ mice. The study also demonstrated reduction in macrophage MCP1 release leading to reduced inflammatory plaques and increased reverse cholesterol transport as a possible mechanism of protection [75]. Recently intranasal immunization with ApoB100 peptides was found to induce protective immune response mediated by antigen-specific Treg and could confer protection against the disease in animal models. Our study showed a synergistic effect of immunization with a combination of ApoB and HSP60 peptides in preventing early atherosclerosis [106]. Molecular mimicry between PC of Ox-LDL and apoptotic cells and that of pneumococcus leading to generation of cross-reactive antibodies which can block uptake of LDL by macrophages has been reported [73]. Thus, immunizations with both ApoB peptides as well as oxidized phospholipids have atheroprotective effects in animal models of diet-induced atherosclerosis.

## 9. Conclusion

The search for alternative and more specific ways to reduce the modification of LDL, which would consequently reduce the immune response to modified lipoproteins, has received continued attention from the atherosclerosis research community. Different epitopes on Ox-LDL is shown to determine its protection or pathogenicity by various studies. The possibility of using Ox-LDL as an immunogen for modulation of response without having any serious adverse effects has to be elucidated. Anti-Ox-LDL antibodies with neutralizing activity against modified LDL may be an effective therapeutic molecule and needs to be deliberated carefully. Another important criterion for a vaccine is to elucidate its potential in preventing the progression of an established plaque. Atherosclerosis is a slow progressing disease in human. Traces of fatty streaks are also observed in children though the disease manifests itself at a much later age. Development of a proper clinical protocol, establishment of surrogate markers, and identification of right patient population to study the vaccine efficacy are some of the important criteria to be explored in the development of a therapeutic vaccine. Careful consideration of these aspects will be most essential before they can be taken as candidates for therapeutic intervention.
